# miR-542-3p overexpression is associated with enhanced osteosarcoma cell proliferation and migration ability by targeting Van Gogh-like 2

**DOI:** 10.3892/mmr.2014.2777

**Published:** 2014-10-27

**Authors:** HUAZHUANG LI, HONGTAO LIU, JINGFANG PEI, HAIYAN WANG, HONGLIN LV

**Affiliations:** 1 Department of Orthopedics, Yidu Central Hospital, Weifang Medical College, Weifang, Shandong 262500, P.R. China; 2 Department of Spinal Orthopedics, Yantai Yuhuangding Hospital, Medical College of Qingdao University, Yantai, Shandong 264000, P.R. China; 3 Central Laboratory, Yantai Yuhuangding Hospital, Medical College of Qingdao University, Yantai, Shandong 264000, P.R. China

**Keywords:** osteosarcoma, microRNA, Van Gogh-like 2, 3′ untranslated region, proliferation, migration

## Abstract

Osteosarcoma is the most common histological form of primary bone cancer, which arises from osteoid tissue. It occurs predominantly in infants and adolescents, with an incidence of 4–5 cases/100,000,000. The 5-year survival rate of patients with osteosarcoma has significantly improved over time; however, there remains a significant proportion of patients that respond poorly to chemotherapy. An improved understanding of the pathology of osteosarcoma is required to provide more effective treatment strategies, identify biomarkers and develop novel chemotherapeutic agents. Disturbance in microRNA (miRNA) expression has been identified in osteosarcoma tissues and cell lines; however, the roles of miRNA during osteosarcoma pathogenesis remain to be elucidated. In the present study, the expression levels of eight selected miRNAs were investigated in osteosarcoma tissues and the results revealed that the expression levels of miR-542-3p and miR-542-5p were significantly upregulated and the expression of miR-199-3p was significantly downregulated. Using a dual luciferase assay and western blot analysis, the present study confirmed that Van Gogh-like 2, which is a non-canonical Wnt pathway suppressor, was a target gene of miR-542-3p. Subsequently, the biological function of miR-542-3p in U2OS cells was examined, which revealed that overexpression of miR-542-3p can enhance the cell proliferation and migration ability of U2OS cells. This indicated that miR-542-3p may act as an oncogene in osteosarcoma pathogenesis. The findings of the present study may provide assistance in understanding the development of osteosarcoma and aid in the development of strategies for the diagnosis and treatment of osteosarcoma.

## Introduction

Osteosarcoma is the most common type of primary bone malignancy, which arises from osteoid tissue and produces immature bone. Osteosarcoma occurs mainly in infants and adolescents and has an incidence of 4–5 cases/100,000,000 (1,2). There has been a significant improvement in the 5 year survival rate of patients with osteosarcoma to ~60–70% since combinatorial chemotherapy was introduced (3). However, the response to chemotherapy is poor in a significant proportion of these patients, with the possibility of local relapse or distant metastasis following curative resection of the primary tumor and intensive chemotherapy (1–4). An increased understanding of the pathogenesis of osteosarcoma is required to improve treatment strategies, identify novel biomarkers and develop chemotherapeutic agents.

MicroRNA (miRNA) is a type of short non-coding RNA, which suppresses the expression of protein coding genes by partial complementary binding, particularly to the 3′ untranslated regions (UTRs) of messenger RNAs (5,6). Alterations to the expression of miRNAs are involved in the initiation, progression and metastasis of human cancer and it is hypothesized that miRNAs function as tumor suppressors and as oncogenes in cancer development (4,5). Several studies have investigated the expression profile of osteosarcoma tissues and a variety of miRNA expression has been identified. Taulli *et al* (6) reported that, in mice xenografts, myogenic differentiation is promoted by the miRNAs, miR-1 and miR-206 to regulate skeletal muscle development and inhibit rhabdomyosarcoma tumor growth. Subramanian *et al* (7) examined the miRNA expression profiles in histological soft tissue samples, including 27 from synovial sarcoma, liposarcoma, rhabdomyosarcoma, leiomyosarcoma and gastrointestinal stromal tumor and seven from normal tissues. In addition, analyses of the miRNA expression profile of 19 human osteosarcoma cell lines by Namløs *et al* revealed 177 miRNAs that were differentially expressed in osteosarcoma cell lines compared with normal bone cells (8).

In order to contribute to the clarification of the roles of miRNA during osteosarcoma pathogenesis, the expression of eight candidate miRNAs was detected in a total of 13 paired soft tissue sarcoma and normal tissue samples in the present study. Following identification of significantly altered miRNAs in a screen, one of their target genes, which was predicted by bioinformatics tools, was selected for studying its function in the migration and invasion ability of U2OS cells.

## Materials and methods

### Patients and tissue samples

The present study had been permitted by the Yidu Central Hospital (Weifang, China) and Yantai Yuhuangding Hospital (Yantai, China). Written informed consent had been obtained from all patients prior to participation in the study. According to the ethical and legal standards [NO. (2011)103 ethical and legal standards of Yantai Yuhuangding Hospital], all specimens were made anonymous. Thirteen pediatric patients who were diagnosed with osteosarcoma were 10–16 (median 13) years old. Prior to neoadjuvant therapy, the tumor biopsies were obtained, freshly frozen and stored at −80°C, and histologically confirmed by pathologists. Osteosarcoma tumor tissue and the corresponding normal bone tissue samples from the same specimens were successively obtained in Yidu Central Hospital or Yantai Yuhuangding Hospital in 2011 and 2012.

### Quantitative reverse-transcription polymerase chain reaction (RT-qPCR)

RT-qPCR analysis was used to determine the relative expression level of eight candidate microRNAs (13). TRIzol reagent (Invitrogen Life Technologies, Carlsbad, CA, USA; 1 ml TRIzol to 50–100 mg of tissue), was used to extract total RNA from the osteosarcoma or normal bone tissues, according to the manufacturer’s instructions. The expression levels of eight candidate miRNAs were measured by TaqMan miRNA RT-qPCR. Single-stranded cDNA for each miRNA was synthesized with TaqMan MicroRNA Reverse Transcription kit (Applied Biosystems, Foster City, CA, USA) referring to the manufacturer’s instructions. TaqMan Universal PCR Master mix with miRNA-specific TaqMan MGB probes (Applied Biosystems, Foster City, CA, USA) was used to amplify the cDNA. U6 snRNA served as a normalizer. Primer sequences were as follows: miR-542-3p forward, 5′-TGT GAC AGA TTG ATA ACT-3′ and stem-loop RT primer, 5′-GTC GTA TCC AGT GCA GGG TCC GAG GTA TTC GCA CTG GAT ACG ACC TGC GGT TTC AGT-3′; miR-21 forward, 5′-TAG CTT ATC AGA CTG AT-3′ and stem-loop RT primer, 5′-GTC GTA TCC AGT GCA GGG TCC GAG GTA TTC GCA CTG GAT ACG ACC TGC GGT CAA CAT-3′; miR-34a forward, 5′-TGG CAG TGT CTT AGC T-3′ and stem-loop RT primer, 5′-GTC GTA TCC AGT GCA GGG TCC GAG GTA TTC GCA CTG GAT ACG ACC TGC GGA CAA CCA-3′; miR-125a forward, 5′-TCC CTG AGA CCC TTT AA-3′ and stem-loop RT primer, 5′-GTC GTA TCC AGT GCA GGG TCC GAG GTA TTC GCA CTG GAT ACG ACC TGC GGT CAC TGG-3′; miR-132 forward, 5′-ACC GTG GCT TTC GAT TG-3′ and stem-loop RT primer, 5′-GTC GTA TCC AGT GCA GGG TCC GAG GTA TTC GCA CTG GAT ACG ACC TGC GGC GAC CAT-3′; miR-143 forward, 5′-TGA GAT GAA GCA CTG T-3′ and stem-loop RT primer, 5′-GTC GTA TCC AGT GCA GGG TCC GAG GTA TTC GCA CTG GAT ACG ACC TGC GGG AGC TAC-3′; miR-199-3p forward, 5′-CCC AGT GTT TAG ACT A-3′ and stem-loop RT primer, 5′-GTC GTA TCC AGT GCA GGG TCC GAG GTA TTC GCA CTG GAT ACG ACC TGC GGG AAC AGA; miR-542-5p forward, 5′-TCG GGG ATC ATC ATG TCA-3′ and stem-loop RT primer, 5′-GTC GTA TCC AGT GCA GGG TCC GAG GTA TTC GCA CTG GAT ACG ACC TGC GGT CTC GTG-3′; common reverse, 5′-GTG CAG GGT CCG AGG T-3′. The scramble control was the result of the random rearrangement of miR-542-3P CAA UAG UUA GAC AGU and the sequence was GAC UAG UCA AGA UUA. Total RNA (1 μg) was used for RT-qPCR. PCR reaction conditions were as follows: Stage 1, 95°C for 15 sec; stage 2 (40 circles), 95°C for 5 sec, 64°C for 34 sec; stage 3, melting curve. All the above experiments were performed in triplicate and repeated three times. Data analysis was performed using Microsoft Excel (Microsoft Corp., Redmont, WA, USA).

### Cell culture

The U2OS cells (Cell Bank of Type Culture Collection of Chinese Academy of Sciences, Shanghai, China) were cultured in Dulbecco’s modified Eagle’s medium (DMEM) containing 10% fetal bovine serum (HyClone, Logan, UT, USA), 100 IU/ml penicillin and 10 mg/ml streptomycin (HyClone, Logan, UT, USA). All cells were maintained at 37°C under an atmosphere of 5% CO_2_.

### 3′-UTR luciferase reporter assays

Luciferase reporter assays were conducted according to a published procedure (14). The target sequence was amplified by PCR using the following primers: Forward, 5′-CCG GTA CCG CTG AAT AGA TCC CTG AGG T-3′ and reverse, 5′-CGC TCG AGG GGC CAG CAA ATT TTG CTC A-3′ and cloned into pGL3-control vector through KpnI and XhoI sites. Mimic was synthesized according to the sequence of miR-542-3p. A VNAGL2 3′-UTR segment (516 bp) containing the predicted miR-542-3p binding site was cloned into the pGL3-control vector (Promega Corporation, Madison, WI, USA) downstream of the firefly luciferase gene, after which the 3′-UTR luciferase reporter was obtained. The control was a mutant target site of miR-542-3p in 3′-UTR of VANGL2. The miR-542-3p mimic and miR-542-3p inhibitor used in this study were customized and synthesized by GenePharma Co., Ltd. (Shanghai, China). Thymidine kinase promoter-*Renilla* luciferase reporter plasmid (pRL-TK; Promega) was co-transfected into the U2OS cells. The cells were seeded in 48-well plates and cultivated at 37°C. Together with the miR-542-3p mimic or the miR-542-3p inhibitor, the luciferase reporter vectors (100 ng) were co-transfected in the presence of 0.5 μl Lipofectamine 2000 (1 μg/ml; Invitrogen Life Technologies) and then incubated for 48 h in 5% CO_2_ at 37°C. Two days following transfection, the cells were collected and evaluated by Dual-Luciferase assay (Promega). Every treatment was performed in triplicate, and independent experiments were performed. The results were presented as the relative luciferase activity, and *Renilla* luciferase was used to normalize the expression of firefly luciferase.

### Western blot analysis

The protein extracts obtained from the cells of osteosarcoma tissue of normal bone tissue samples were boiled in SDS/β-mercaptoethanol sample buffer [containing Tris–HCl, SDS, β-mercaptoethanol, bromophenol blue and glycerol (Owl Scientific, Inc., Woburn, MA, USA)] at 55°C for 15 min, and 20 μg of each extract was loaded into each lane of 8% polyacrylamide gels. The proteins were separated by electrophoresis and the proteins in the gels were blotted onto polyvinylidene difluoride membranes (Amersham Pharmacia Biotech, St. Albans, UK) by electrophoretic transfer. The membrane was incubated with goat anti-VANGL2 polyclonal antibody (Abcam, Cambridge, MA, USA) and mouse anti-β-actin monoclonal antibody (Santa Cruz Biotechnology, Inc., Santa Cruz, CA, USA) for 1 h at 37°C. The specific protein-antibody complex was then detected using horseradish peroxidase-conjugated rabbit anti-goat or rabbit anti-mouse immunoglobulin (Ig)G (Cell Signaling Technology, Inc., Danvers, MA, USA). After the membrane was washed using PBS containing 0.1% Triton-X 100), the determinands were developed using an enhanced chemiluminescence kit (ECL; Pierce Biotechnology, Inc., Appleton, WI, USA) and exposed to an X-ray film (X-Omat XBT-1; Kodak, Rochester, NY, USA) for 1 min. The film was quantified using ImageJ software (version 2.1.4.7; National Institutes of Health, Bethesda, MD, USA). β-actin signal was used as a loading control.

### Cell proliferation assay

The U2OS cells were seeded into 96-well plates at low density (5×10^3^ cells/well) in DMEM culture overnight for attachment to occur. The cells were then transfected with either the miR-542-3p mimic or the scrambled miRNA (miRNA control). MTT (5 mg/ml; 20 μl; Sigma, St. Louis, MO, USA) were added into each well 48 h after transfection and the cells were incubated for a further 4 h. The absorbance was recorded at A570 nm using a 96-well plate reader (BioTek Instruments, Inc., Burlington, VT, USA) following the addition of 150 μl dimethyl sulfoxide (Sigma).

### In vitro migration assays

The U2OS cells transfected with the miR-542-3p mimic, scramble miRNA, miR-542-3p inhibitor or anti-miR control were harvested 48 h after transfection and subjected to the following assays. For migration assays, the transfected cells (0.5×10^6^ cells/ml) were seeded into the top of an 8.0-mm pore membrane chamber (Corning Costar, Cambridge, MA, USA). Following 12 h of incubation, the cells that had passed through the membrane to attach to the bottom of membrane were fixed with methanol and stained with hematoxylin and eosin (Sigma). The cells were scraped and removed from the top of chamber, the membranes were mounted on cover slides and the cell migration was quantified by counting the number of cells that had passed through the pores in five randomly selected fields per sample at magnification of ×100 under a microscope (Nikon Eclipse TE2000-U; Nikon, Tokyo, Japan).

### Statistical analysis

Data were analyzed using SPSS statistical software, version 16 (SPSS, Inc., Chicago, IL, USA) followed by analysis with the independent two-sample t-test. P<0.05 was considered to indicate a statistically significant difference.

## Results

### Disturbance of the microRNA expression profile in osteosarcoma tissues

There is evidence that altered patterns of miRNA expression correlate with various human diseases and particularly several types of cancer (7,8). The behavior of miRNAs is complex due to their regulation of hundreds of targets, which can result in the downregulation of numerous target genes, including oncogenes and tumor suppressor genes. Therefore, examining their clinical potential is particularly worthwhile.

**Figure 1:**
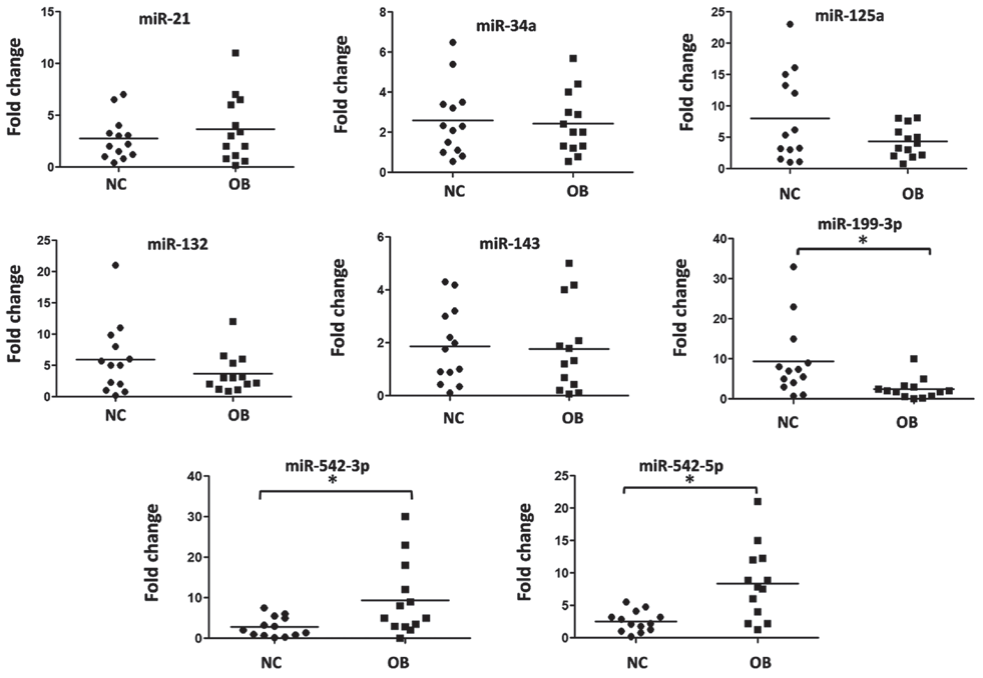
Disturbed miRNA expression in osteosarcoma tissue samples. The expression level of eight candidate miRNAs was detected in individual samples using TaqMan miRNA reverse transcription-quantitative polymerase chain reaction. Statistical analyses were performed to analyze the overall trend of each miRNA in all osteosarcoma tissue samples. U6 was used as an internal reference among the different samples and to normalize for experimental error. ^*^P<0.05. miRNA and miR, microRNA; NC, normal control; OB, osteosarcoma.

Several studies have reported that the miRNA expression profile is altered significantly in the progression of osteosarcoma (9,10), however, further clarification is required. In the present study, the expression profiles of eight miRNAs, which were identified by another study as differentially expressed in osteosarcoma tissues or osteosarcoma cell lines (11), were determined by RT-qPCR. As shown in Fig. 1, miR-542-3p and miR-542-5p were significantly upregulated and miR-199-3p was significantly downregulated in the osteosarcoma tissues.

### VANGL2 expression is repressed by miR-542-3p

The function of an miRNA is reflected mainly in its repressive effects on the expression of its target genes (12,13). miR-542-3p and miR-542-5p are products of the same molecule, pre-miR-542, and miR-542-5p was considered to be the passenger strand, therefore the present study investigated the target gene of miR-542-3p using online bioinformatics tools. Using miRanda (http://www.microrna.org/microrna/home.do) and TargetScan (http://www.targetscan.org/; and Pictar, http://pictar.mdc-berlin.de/), VANGL2 was identified as a possible target gene of miR-542-3p, the downregulation of which is associated with enhanced cancer cell migration and invasion ability.

**Figure 2:**
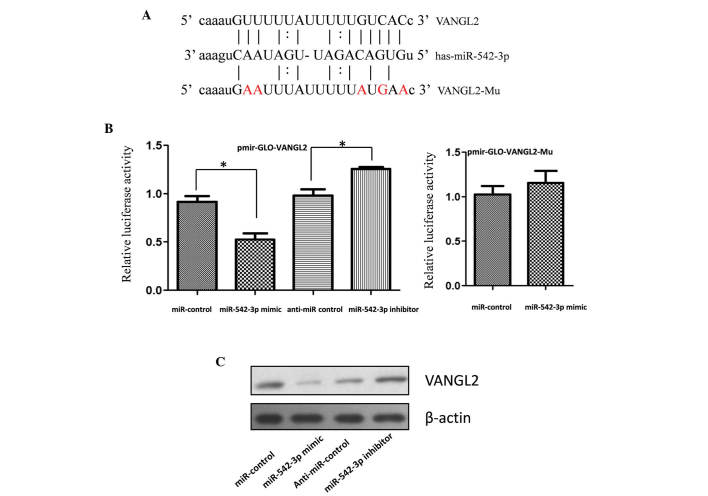
Expression of VANGL2 is suppressed by miR-542-3p. (A) Schematic diagram for constructing the predicted miR-542-3p binding site into the pGL3 control vector. (B) Confirmation of the miR-542-3p target gene. U2OS cells were co-transfected with the miRNA control, miR-542-3p mimic, anti-miR control or miR-542-3p inhibitor and pGL3-VANGL2 for the dual-luciferase assay. PRL-TK, containing RL, was cotransfected with the 3′-UTR of VANGL2 for data normalization. (C) Mutation analysis of the miR-542-3p binding site. Following mutation of five nucleotides of the binding site of miR-542-3p in the 3′-UTR of VANGL2 (pGL3-VANGL2-Mu), the luciferase activity was significantly decreased in the U2OS cells cotransfected with the miR-542-3p mimics and pGL3-VANGL2 compared with those cotransfected with pGL3-VANGL2-Mu or pGL3. (D) VANGL2 protein levels in the miR-542-3p mimic and inhibitor-treated U2OS cells were detected by western blot analysis. miR, microRNA; VANGL2, Van Gogh-like 2; UTR, untranslated region; Mu, mutant; PRL-TK, thymidine kinase promoter-Renilla luciferase reporter plasmid.

To confirm whether VANGL2 was the target gene of miR-542-3p, a 516 bp segment of the VANGL2 3′-UTR, containing the interaction sites of miR-542-3p, was cloned into the pGL3 control vector (pGL3-VANGL2) downstream of the firefly luciferase reporter gene (Fig. 2A) to perform a dual luciferase assay. The U2OS cells were cotransfected with pGL3-VANGL2 and either the miR-542-3p mimic or inhibitor (Fig. 2B). Compared with the miRNA control, luciferase activity was significantly decreased by miR-542-3p by ~47.8% (P<0.05). Furthermore, luciferase activity was significantly increased by the miR-542-3p inhibitor compared with the anti-miR control by ~23.5% (P<0.05). These results indicated that miR-542-3p targets the 3′-UTR of VANGL2, leading to a change in firefly luciferase translation.

A seed sequence mutation clone was also used to confirm the binding site for miR-542-3p (Fig. 2A). A vector, containing putative miR-542-3p binding regions in the 3′-UTR of VANGL2 with five mutant nucleotides (pGL3-VANGL2-Mu) was used and a wild type VANGL2 vector was used as a control. As the histogram in Fig. 2B (right) shows, there were no differences in enzyme activity between the cells cotransfected with miR-542-3p mimics or pGL3-VANGL2-Mu compared with pGL3-VANGL2. These data indicated that miR-542-3p may suppress the expression of VANGL2 by binding to the seed sequence at the 3′-UTR of VANGL2.

### miR-542-3p regulates the endogenous expression of VANGL2 in U2OS cells

Although VANGL2 was identified as a target gene for miR-542-3p, whether miR-542-3p was able to regulate the endogenous expression of VANGL2 remained unclear. The U2OS cells were transfected with the miR-542-3p mimic or inhibitor to determine whether dysregulation of the expression of miR-542-3p affected the endogenous expression of VANGL2. Compared with the corresponding control, the protein expression of VANGL2 was significantly suppressed by the miR-542-3p mimics and upregulated by the miR-542-3p inhibitor (Fig. 2C).

### miR-542-3p overexpression enhances U2OS cell viability

To determine the possible function of miR-542-3p in the pathogenesis of osteosarcoma, the effect of miR-542-3p on the growth of osteosarcoma cells was detected using an *in vitro* cell line model.

**Figure 3:**
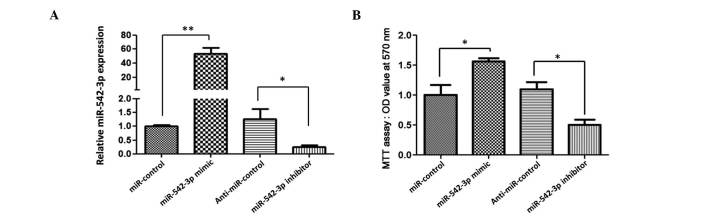
U2OS cells are enhanced by miR-542-3p. (A) Reverse transcription-quantitative polymerase chain reaction was used to detect the expression of miR-542-3p in the transfected U2OS cells. (B) An MTT assay was used to detect cell proliferation ability. miR, microRNA; OD, optical density.

The U2OS cells were transfected with either the miR-542-3p mimic, miR control, miR-542-3p inhibitor or anti-miR control. The results demonstrated that the expression level of miR-542-3p in the U20S cells was increased in the cells transfected with the miR-542-3p mimic compared with the pre-miR control (P<0.01) and was decreased in the cells transfected with the miR-542-3p inhibitor compared with the anti-miR control (P<0.05) as shown in Fig. 3A.

Cell proliferation and viability were determined using an MTT assay 48 h after transfection. As shown in Fig. 3B, the relative proliferation rates in the U2OS cells transfected with miR-542-3p mimics were increased by ~52.3% compared with the pre-miR control (P<0.05). The relative proliferation rates in the U2OS cells transfected with the miR-542-3p inhibitor decreased ~55.2% compared with the anti-miR control (P<0.05). These results indicated that overexpression of miR-542-3p significantly increased osteosarcoma cell viability, while downregulation of miR-542-3p repressed osteosarcoma cell proliferation.

### miR-542-3p modulates the migration capacity of osteosarcoma cells in vitro

**Figure 4:**
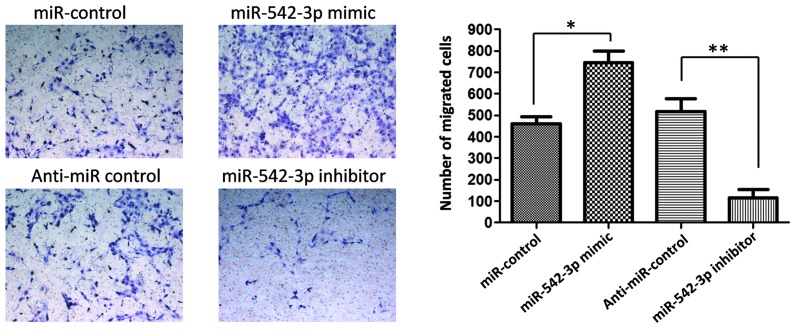
miR-542-3p enhances U2OS cell migration. U2OS cells were transfected with either the pre-miR control, miR-542-3p mimic, anti-miR control or miR-542-3p inhibitor. The cells were harvested 48 h after transfection, recounted to 0.5×10^6^ cells/ml in every group and seeded into Transwells for a cell migration assay. Subsequently, the cells on the top of the membranes were removed and the cells on the bottom of the membranes were stained with hematoxylin and eosin. Cell migration was quantified by counting the number of cells passing through the membrane from five randomly selected fields in each sample 12 h after incubation (magnification, ×100). Representative photomicrographs of the cells passing through the membrane (magnification, ×100) are shown. Data are expressed as the mean of independent triplicate experiments. ^*^P<0.05; ^**^P<0.01. miR, microRNA.

In order to investigate the role of miR-542-3p in the metastasis of osteosarcoma cells, the present study then analyzed the effects of miR-542-3p on the migratory behavior of osteosarcoma cells (Fig. 4). The results revealed that the migration capacity of the U2OS cells transfected with the miR-542-3p mimic were significantly higher compared with those transfected with the miR control (P<0.05). Conversely, the migration capacity was significantly suppressed in the U2OS cells transfected with the miR-542-3p inhibitor compared with the anti-miR control (P<0.01). These findings suggested that the level of miR-542-3p may be closely associated with the metastasis of osteosarcoma cells.

## Discussion

Previous evidence has demonstrated that altered patterns of miRNA expression are associated with various human diseases and, in particular, several types of cancer. The behavior of miRNAs is complex as they regulate hundreds of targets, which can result in the downregulation of numerous genes, including oncogenes and tumor suppressors. Therefore, examining the clinical potential of miRNAs is particularly useful.

In the present study, the expression of eight candidate miRNAs was detected in 13 soft tissue sarcoma samples and 13 normal tissue samples by RT-qPCR. The miRNAs miR-542-3p and miR-542-5p were significantly upregulated and miR-199-3p was significantly downregulated. As miR-542-3p and miR-542-5p are products of pre-miR-542, of which miR-542-3p is the main product, the present study subsequently investigated the biological function of upregulated miR-542-3p. The possible target genes of miR-542-3p were predicted using online bioinformatics tools. Of the thousands of predicted miR-542-3p target genes, the expression of VANGL2 was found to be repressed by miR-542-3p. VANGL2 belongs to the non-canonical WNT pathway, whose activation inhibits canonical WNT signaling. Previous studies have demonstrated that the expression of VANGL2 is significantly downregulated in several cancer cell lines and primary tumors, and a low expression of VANGL2 is associated with a significantly worse clinical outcome in neuroblastoma (9–11). Additionally, siRNA experiments have revealed that knock down of VANGL2 increases cell proliferation of neuroblastoma cells (10). Wnt transduction pathways function to modulate cell fate and proliferation, and regulate cell growth and differentiation in a variety of organ systems (16). In osteosarcoma cells, as opposed to normal cells, several Wnt ligands, receptors and coreceptors are highly expressed, while Wnt inhibitors are downregulated; therefore, Wnt signaling is aberrantly activated (17). VANGL2 has been proved to be structurally associated with the Wnt pathway, and to be implicated in the development of osteosarcomas (18). However, as a target gene of miR-542-3p, whether the osteosarcoma cell proliferation and migration mediated by VANGL2 are regulated by miR-542-3p has remained elusive. Therefore, the present study investigated the impact of miR-542-3p overexpression on U2OS cell proliferation and migration ability. As expected, miR-542-3p enhanced U2OS cell proliferation and migration, which indicated that miR-542-3p may act as an oncogene by repressing VANGL2 and, thus, preventing the inhibition of the non-canonical WNT pathway.

In conclusion, the results of the present study indicated that miR-542-3p may function as an oncogene by targeting VANGL2 during osteosarcoma pathogenesis. These findings may provide insight into the development of osteosarcoma and aid in the development of novel diagnostic and therapeutic strategies for osteosarcoma.
